# Privileged Scaffold Decoration for the Identification of the First Trisubstituted Triazine with Anti-SARS-CoV-2 Activity

**DOI:** 10.3390/molecules27248829

**Published:** 2022-12-12

**Authors:** Silvia Cesarini, Ilaria Vicenti, Federica Poggialini, Massimiliano Secchi, Federica Giammarino, Ilenia Varasi, Camilla Lodola, Maurizio Zazzi, Elena Dreassi, Giovanni Maga, Lorenzo Botta, Raffaele Saladino

**Affiliations:** 1 Department of Biological and Ecological Sciences, University of Viterbo, Via S.C. De Lellis s.n.c., 01100 Viterbo, Italy; 2 Department of Medical Biotechnologies, University of Siena, 53100 Siena, Italy; 3 Department of Biotechnology, Chemistry, and Pharmacy (DBCF), University of Siena, 53100 Siena, Italy; 4 Institute of Molecular Genetics, IGM CNR “Luigi Luca Cavalli-Sforza”, Via Abbiategrasso 207, 27100 Pavia, Italy

**Keywords:** SARS-CoV-2, DDX3X, privileged scaffold, s-triazines, decoration approach, antivirals

## Abstract

Current therapy against severe acute respiratory syndrome coronavirus type 2 (SARS-CoV-2) are based on the use of Remdesivir **1**, Molnupiravir **2**, and the recently identified Nirmatrelvir **3**. Unfortunately, these three drugs showed some limitations regarding potency and possible drug–drug interactions. A series of derivatives coming from a decoration approach of the privileged scaffold s-triazines were synthesized and evaluated against SAR-CoV-2. One derivative emerged as the hit of the series for its micromolar antiviral activity and low cytotoxicity. Mode of action and pharmacokinetic in vitro preliminary studies further confirm the role as candidates for a future optimization campaign of the most active derivative identified with this work.

## 1. Introduction

**Figure 1:**
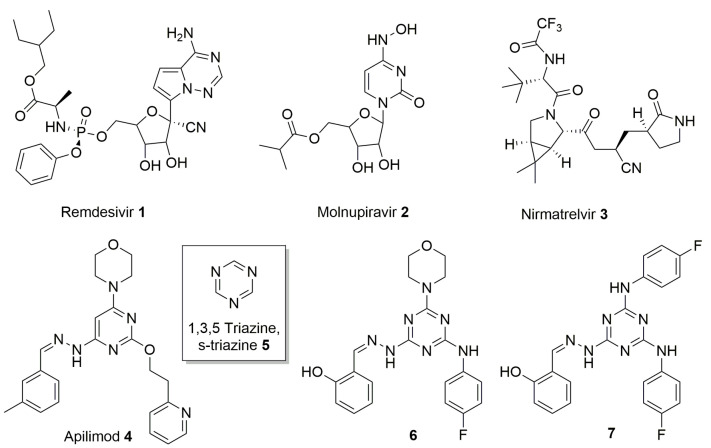
Chemical structures of Remdesivir **1**, Molnupiravir **2,** Nirmatrelvir **3**, Apilimod **4**, s-triazine **5,** and two highly decorated triazines previously identified **6** and **7**.

Severe acute respiratory syndrome coronavirus type 2 (SARS-CoV-2) is a positive-strand RNA virus [1], belonging to the family of Corona viridae, discovered in late 2019 [2]. It is the causative agent of the coronavirus disease 2019 (COVID-19), that led to the pandemic outbreak and the consequent global lock-down measures of virus containment in 2020. Severe diseases in animals and in humans are associated with COVID-19 at gastrointestinal and, more importantly, respiratory level, with severe pneumonia, high fever, dry cough, and difficulty in breathing as common symptoms at the onset of illness [3]. Worldwide, vaccination has been the main method to reduce the spread of infection and the severity of disease in healthy individuals. In SARS-CoV-2 infected patients at high risk of disease progression, different therapeutic agents targeting directly the viral replication cycle are currently available [4,5], including different monoclonal antibodies, antiviral drugs repurposed, such as Remdesivir **1** (FDA approved) and Molnupiravir **2** (authorized under emergency use authorization, EUA), or recently identified, such as Nirmatrelvir **3** (EUA) (Figure 1). However, the development of novel drugs remains a key priority because currently available antivirals are not highly effective, and their use is limited by a number of drug–drug interactions (Nirmatrelvir), by inconvenient administration (monoclonal antibodies and Remdesivir) or by the toxicity in fragile population (Molnupiravir) [6].

1,3,5-Triazine **5**, known as symmetric triazine (s-triazine), is an extensively studied heterocyclic nucleus in medicinal chemistry for its easy chemical manipulation and its wide therapeutic application (Figure 1) [7]. s-Triazine is considered, in fact, a privileged structure [8] since it is the core structure of antibacterial, antiviral, anticancer, and antifungal agents [9].

In the context of s-triazine-based compounds, in 2011, our group reported the synthesis of a series of derivatives presenting this privileged core structure, as inhibitor of the human DEAD-box RNA helicase DDX3X, endowed with anti-HIV activity [10]. DDX3X is a potential target for the development of anticancer and antiviral compounds considering its oncogenic role in promoting cancer progression and its active involvement in the replication of different viruses [11,12,13]. From the library of compounds synthesized, two members (compounds **6** and **7**, Figure 1) emerged for their antiretroviral effect in the low-micromolar range (EC_50_ = 2.5 and 2.0 μM, respectively), even if accompanied by a moderate cytotoxicity (CC_50_ = 10.0 and 8.0 μM, respectively) in peripheral blood mononuclear cells (PBMC).

**Scheme 1:**
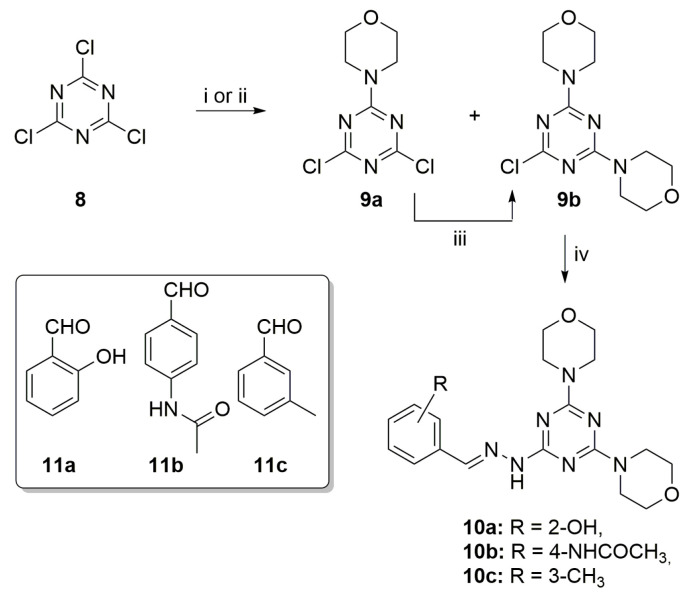
Synthesis of triazine derivatives **10a–c**. Reagents and conditions: (i) DME, −60 °C, morpholine, 5 h; (ii) *N,N*-Diisopropylethylamine (DIPEA), morpholine, EtOH, 25 °C, 18 h; (iii) CH_2_Cl_2_, 25 °C, morpholine, 18 h; (iv) 1. CH_2_Cl_2_, NH_2_NH_2_, reflux 12 h, 2. toluene, **11a–c**, 3 h, reflux, Dean–Stark.

Driven by our interest in the functionalization of privileged scaffolds [14] and by the possibility of developing novel antiviral agents, we decided to obtain and to test s-triazine-based compounds, starting from **6** and **7**, against the emerging SARS-CoV-2. Herein, is described the synthesis and the biological evaluation of a focused library of nine highly decorated triazines as inhibitor of the above-mentioned coronavirus. In particular, substituents with precise functions have been introduced on the azine core to investigate the chemical space around the privileged nucleus. Salicyl aldehyde, (Scheme 1, compound **11a**) and 4-fluoro aniline were chosen due to the high antiretroviral activity showed by derivatives **6** and **7**. In addition, 4-acetamidobenzaldehyde (Scheme 1, compound **11b**) was selected on the basis of the inhibitory activity on DDX3X of some 4-acetamido-phenyl-triazines presented in our previous work [10]. Morpholine was chosen on the basis of the activity of **6** and **7** and also for its capacity to improve drug-like and pharmacokinetic properties [15]. Finally, 3-tolualdehyde (Scheme 1, compound **11c**) was used to reproduce a pharmacophoric moiety present on Apilimod (Figure 1, compound **4**), a kinase inhibitor repurposed toward SARS-CoV-2 [16] and structurally similar to the hit compounds **6** and **7**, already identified. In addition, in vitro absorption, distribution, metabolism, and excretion (ADME) tests were conducted on the most active compound to further confirm its positive physical chemical properties and its potential role as optimizable hit compound.

## 2. Results and Discussion

A series of three highly decorated triazine derivatives **10a–c**, was synthesized starting from 2,4,6-trichloro-1,3,5-triazine (cyanuric chloride) **8** following the synthetic procedure reported in Scheme 1.

Compound **8** (1.0 mmol) was reacted with morpholine (1.0 mmol) at −60 °C in dimethoxymethane as solvent, affording a mixture of the monosubstituted **9a** and disubstituted **9b** intermediates, isolated after flash column chromatography with a 55% and 15% yield, respectively. Attempts to obtain only compound **9a**, such as decreasing the temperature to −78 °C or the use of a lower amount of morpholine (0.7 mmol instead of 1.0 mmol) failed. On the other hand, it was possible to directly afford compound **9b** using a higher amount (2.2 mmol) of morpholine at 25 °C or reacting the latter (2.0 mmol) with **9a** in CH_2_Cl_2_ at 25 °C. Subsequently, from the disubstituted intermediate **9b,** the three final compounds **10a–c** can be synthesized by displacement of the third chlorine atom with hydrazine, and reductive amination with the desired aromatic aldehydes **11a–c** to form an imine linkage with a 50, 25, and 45% yield, respectively.

**Scheme 2:**
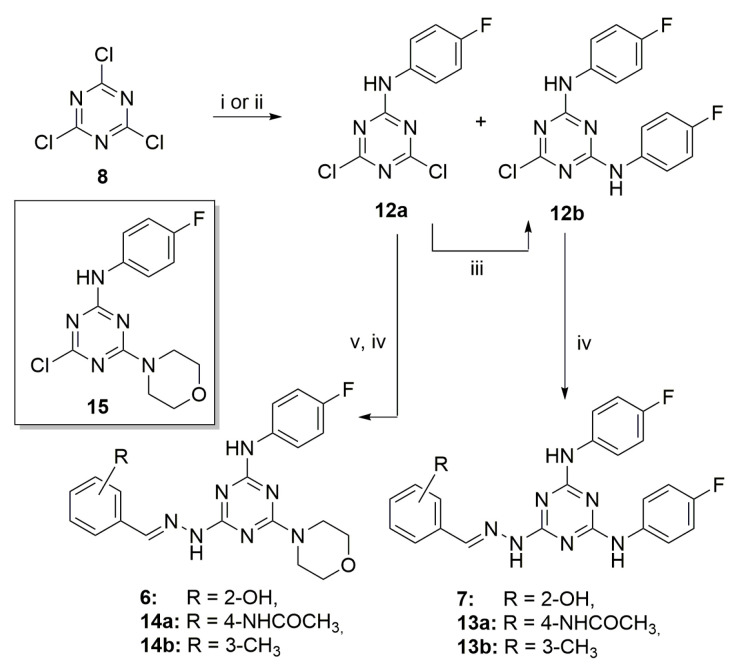
Synthesis of triazine derivatives **6**, **7**, **13a–b** and **14a–b**. Reagents and conditions: (i) DME, −60 °C, 4-fluoroaniline, 5 h; (ii) DIPEA, 4-fluoroaniline, EtOH, 25 °C, 18 h; (iii) CH_2_Cl_2_, 25 °C, 4-fluoroaniline, DIPEA 18 h; (iv) 1. CH_2_Cl_2_, NH_2_NH_2_, reflux 12 h, 2. MeOH, **11a–c**, acetic acid, 25 °C, 18 h; (v) CH_2_Cl_2_, 25 °C, morpholine, 5 h.

As depicted in Scheme 2, a closely related pathway was used to synthesize compounds **6** and **7** and the highly decorated triazines **13a–b** and **14a–b**. The reaction of **8** (1.0 mmol) with 4-fluoroaniline (1.0 mmol) at −60 °C or even at −78 °C furnished unselectively compounds **12a** and **12b** with a 47% and 10% yield, after chromatographic purification. The monosubstituted **12a** can be easily converted in the disubstituted **12b** by reaction with 4-fluoroaniline and *N,N*-Diisopropylethylamine (DIPEA) at 25 °C with a 74% yield. Then, **12b** is converted into the final derivatives **7**, **13a–b** by nucleophilic substitution in the presence of hydrated hydrazine and reaction with the opportune aldehyde.

Finally, the monosubstituted derivative **12a** was subjected to aromatic nucleophilic substitution with morpholine to give compound **15** (Scheme 2). From intermediate **15**, the trisubstituted triazines **6**, **14a–b** were synthesized by chlorine displacement with hydrated hydrazine and the addition, in turn, of salicyl aldehyde, 4-acetamidobenzaldehyde, and 3-tolualdehyde **11a–c**, respectively. Full details of the synthetic procedures and characterization data are included in the Supplementary Materials.

The inhibitory activity of the resynthesized compounds and the novel triazines designed against SARS-CoV-2 was analyzed in vitro, using the marketed drug Remdesivir as reference (Table 1). The human colon epithelial carcinoma cell line Caco-2, permissive for SARS-CoV-2 infection [17], was used to determine the cytotoxicity and the anti-SARS-CoV-2 activity of compounds **6**, **7**, **10a–c**, **13a,b**, and **14a,b** as previously described [18] with minor modifications as indicated in the Materials and Methods section below. Briefly, the semi confluent Caco-2 cell line was infected with SARS-CoV-2 at 0.004 Multiplicity of Infection (MOI) in the presence of serial dilutions of the investigational and reference compound (Remdesivir), starting from the non-toxic dose, as previously determined in cytotoxic assay. After 72 h incubation, the antiviral activity of the compounds was measured by immunodetection of the viral N protein in the cell monolayer and expressed as half-maximal inhibitory concentration (IC_50_). The Selectivity Index (SI) of compounds was calculated as the ratio between the half-maximal cytotoxic concentration (CC_50_) and the IC_50_.

Six compounds, namely **10a–c**, **13a,b**, and **14a** showed a low cytotoxicity (Table 1, entries 1–3, 5, 6, and 8, respectively), compared to the derivatives obtained in the previous study, derivatives **6** and **7** (Table 1, entries 4 and 7). These results highlighted the importance of the introduction of other pharmacophoric moieties, namely the 3-tolyl and 4-acetamidophenyl portions, compared to the salicylic one, as well as the presence of two morpholine substituents on the azine core structure. Regarding the antiviral effect, compound **10a** is the only one that highlighted micromolar (12.1 μM) activity against SARS-CoV-2. Even if the activity of **10a** was lower than that reported for Remdesivir, the low cytotoxicity showed by this compound prompted us to further evaluate its pharmacokinetic properties, in the light of possible future chemical optimizations.

In addition, all the novel compounds synthesized were also evaluated for their antiretroviral activity but, surprisingly, they seem to be unable to inhibit HIV replication compared to parent compounds **6** and **7** (Table S1 (Supplementary Materials)).

For the more promising compound, derivative **10a**, the inhibition of the ATPase activity of the human helicase DDX3X was evaluated. Different concentrations of the compound were incubated with 1 µM of purified recombinant DDX3X and ATPase activity was determined with the ADP-glo assay. This assay showed that the highly decorated triazine **10a** is able to inhibit DDX3X with a 6.6 μM ID_50_ value (Figure S1). This result further confirms the association of the antiviral activity with the inhibition of the human helicase already observed for the parent compounds **6** and **7**.

**Figure 2:**
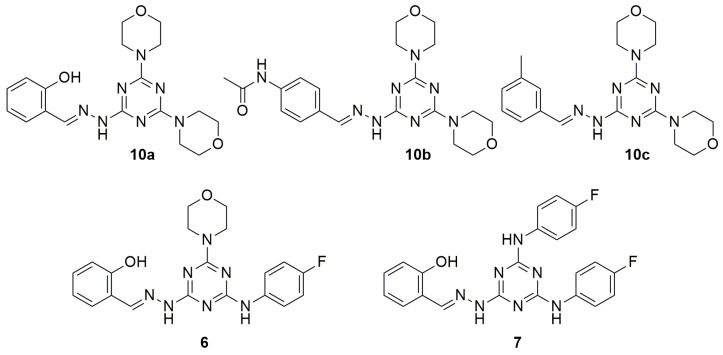
Structure of compounds **6**, **7**, **10a–c**.

In order to perform the structure–activity relationship (SAR) studies, the in vitro ADME profile of compounds **6**, **7**, **10a–c** (Figure 2) was intensively investigated.

Firstly, the water solubility of compounds was studied dissolving 1 mg of solid compound into 1.0 mL of Mill-Q H_2_O. As reported in Table 2, the results obtained suggested a good water solubility for compounds **10a–c**, with LogS values around −5. The replacement of one or two morpholine moieties with para fluorophenyl groups, worsens the solubility of compounds **6** and **7**, to less than 0.1 µg/mL (limit of detection, LOD).

The trend appreciated in terms of solubility was confirmed by results obtained from permeability studies. In fact, parallel artificial membrane permeability assay (PAMPA) underlined how the low solubility of compounds **6** and **7** was accompanied by a major tendency of these derivatives to interact with the lipidic artificial membrane where they probably remained entrapped. Hence, compounds **6** and **7** resulted in being characterized by P_app_ of 4.74 and 4.58 cm/s × 10^−6^ and a general high percentage of membrane retention (MR 28.5% and 15.5%, respectively). Compounds **10a–c**, endowed with a higher water solubility, showed a lower tendency to interact with the lipidic bilayer (MR < 10%) and an improved capability to cross the membrane for passive diffusion (P_app_ from 7.89 to 9.78 cm/s × 10^−6^) (Table 3).

From a metabolic point of view, all compounds resulted in a very high stability (more than 99.9%) when incubated for 1 h in the presence of human liver microsomes (HLM). Finally, further studies were performed in order to investigate the stability in the presence of human plasma. All compounds were incubated at the fixed concentration of 100 µM in plasma for 24 h. As reported in Table 4, all compounds resulted in not being affected by the hydrolytic action of plasma esterase with percentages of plasma stability of 98–99% after 24 h of incubation.

The replacement of one or both the 4-fluoroaniline substituents, of compounds 6 and 7, with morpholine increased the water solubility and positively influenced parallel artificial membrane crossing and membrane retention. Compounds 10a–c, in fact, showed better pharmacokinetic properties compared to 6 and 7. In addition, the presence of two morpholines lead to a decrease in the cytotoxicity. Regarding the aromatic hydrazone moiety, salicyl residue of 10a contributed to the most pronounced decrease in the cytotoxicity and to the anti-SARS-CoV-2 activity. The 4-acetamidophenyl and 3-toluic groups of 10b and 10c, respectively, led to better data of membrane permeability but not to antiviral effects.

## 3. Materials and Methods

### 3.1. Chemistry-General Part

All reactions were performed in flame-dried glassware under a nitrogen atmosphere. Reagents were obtained from commercial suppliers (Merck Srl, Milan, Italy) and used without further purification. TLC chromatography was performed on precoated aluminum silica gel SIL G/UV254 plates (Macherey-Nagel GmbH & Co. Düren, Germany). The detection occurred via fluorescence quenching or development in a ninhydrin solution (0.2 g of ninhydrin in 99.5 mL ethanol and 0.5 mL acetic acid.). Merck silica gel 60 was used for chromatography (23–400 mesh). ^1^H NMR and ^13^C NMR spectra were measured on a Bruker Avance DRX400 (400 MHz/100 MHz) spectrometer. Chemical shifts for protons were reported in parts per million (ppm, *δ* scale) and internally referenced to the deuterated dimethyl sulfoxide (DMSO-*d*_6_), methanol (CD_3_OD) or chloroform (CDCl_3_) signal at *δ* 2.50, 3.33 and 7.28 ppm, respectively. ^1^H-NMR spectra are reported in this order: multiplicity and number of protons. Signals were characterized as: s (singlet); d (doublet); dd (doublet of doublets); t (triplet); m (multiplet); bs (broad signal). Mass spectra were recorded with an Agilent 1100 LC/MSD VL system (G1946C) (Agilent Technologies, Palo Alto, CA, USA).

### 3.2. Biology

#### 3.2.1. Cells and Viruses

The SARS-CoV-2 strain belonging to lineage B.1 (EPI_ISL_2472896) was kindly provided by the Department of Biomedical and Clinical Sciences Luigi Sacco, University of Milan (Italy). Once expanded in VERO E6 (African green monkey kidney-cell line, ATCC catalog. N. CRL-1586), the SARS-CoV-2 viral stock was stored at −80 °C and titrated by plaque assay, as previously described [19]. HIV-1 wild-type reference strain NL4-3 (catalog. n. ARP2006), was obtained through the NIH AIDS Reagent Program and viral titer was calculated in TZM- bl cells through the detection of β-galactosidase expression, as previously described [20].

The Caco-2 adherent cell line (ATCC catalog. n. HTB-37), derived from a human colon carcinoma, was used to determine the cytotoxicity and the antiviral activity of candidate compounds against SARS-CoV-2. The H9 suspension cell line (repository code ARP0001; NIBSC Centre for AIDS reagents) derived from a human T cell lymphoma was used to evaluate compounds against HIV-1 in combination with the TZM-bl (repository code ARP5011, NIBSC Centre for AIDS reagents) adherent cell line as described in the antiviral assays’ section.

Adherent cell lines were propagated in high glucose Dulbecco’s Modified Eagle’s Medium with sodium pyruvate and L-glutamine (DMEM; Euroclone), for TZM-bl, or Minimum Essential Medium Eagle (EMEM; Euroclone), for Caco-2, supplemented with 10% Fetal Bovine Serum (FBS; Euroclone) and 1% Penicillin/Streptomycin (Pen/Strep; Euroclone). The propagation medium with a lower concentration of FBS (1%) was used for viral propagation, cytotoxicity, and antiviral activity experiments in adherent cell lines. Suspension cells were propagated in RPMI 1640 medium supplemented with 10% Fetal Bovine Serum (FBS; Euroclone), 2 mM L-glutamine, and 1% Penicillin/Streptomycin (Pen/Strep; Euroclone). Cells were incubated at 37 °C in a humidified incubator supplemented with 5% CO_2_.

#### 3.2.2. Drugs and Cytotoxicity Assay

The cytotoxicity of investigational compounds was determined by CellTiter-Glo 2.0 Luminescent Cell Viability Assay (Promega) according to the manufacturer’s protocol. Cell viability was calculated by measuring cellular ATP as a marker of metabolically active cells through a luciferase-based chemical reaction. The luminescent signal obtained from cells treated with serial dilution of the investigational compounds, or DMSO as control, was measured through the GloMax^®^ Discover Multimode Microplate Reader (Promega) and elaborated with the GraphPad PRISM software version 9 (La Jolla) to calculate the half-maximal cytotoxic concentration (CC_50_). Remdesivir (MCE^®^ cat. HY-104077) and Raltegravir (MCE^®^ cat. HY-10353), used as reference compounds for SARS-CoV-2 and HIV-1 antiviral tests, respectively, were purchased from MedChem Express and dissolved in water (Raltegravir) or 100% DMSO (Remdesivir). Following the determination of the CC_50_, for each compound a non-toxic dose was chosen and used as the starting drug concentration in the subsequent antiviral assays.

#### 3.2.3. Antiviral Assays-SARS-CoV-2

To determine the antiviral activity of candidate compounds against SARS-CoV-2, a direct-yield reduction assay, based on the infection of cells in the presence of serial drug dilutions, was performed as previously described, with minor modifications. Briefly, Caco-2 cells, pre-seeded in a 96-well format, were infected with SARS-CoV-2 viral stock at 0.004 multiplicity of infection (MOI). After 1h adsorption at 37 °C, the viral inoculum was removed and serial dilutions of each tested compound, starting from the non-toxic dose, were added to the infected cells. After 72 h incubation, the antiviral activity was measured in the cell monolayer by immunodetection assay (IA), as previously described [18].

Absorbance was measured at 450 nm optical density (OD450) using the Absorbance Module of the GloMax^®^ Discover Multimode Microplate Reader (Promega). In each plate was included the corresponding reference compound, the mock control (uninfected cells), the virus control, and the virus back titration, performed by diluting 2-fold the initial viral inoculum. Each IA run was validated when both the OD450 values of virus control and the first 2 dilutions of the virus back titration were above 1 OD450. All drug concentrations were tested in duplicate in two independent experiments and in each plate, Remdesivir was used as a reference compound. Infected and uninfected cells without drugs were used to calculate the 100% and 0% of viral replication, respectively. The half-maximal inhibitory concentration (IC_50_) was calculated through a non-linear regression analysis of the dose–response curves generated with GraphPad PRISM software version 9. The Selectivity Index (SI) of compounds was calculated as a ratio between CC_50_ and IC_50_.

### 3.3. Enzymatic Assay

The ATPase enzymatic activity of DDX3X was determined by the ADP-Glo kit (Promega, MD, USA). The reaction was performed in ATPase buffer (20 mM Tris–HCl pH 8, 2 mM DTT, 70 mM KCl, 2 mM MgCl_2,_ 5% DMSO) pre-incubating 1 µM DDX3X with different concentrations of inhibitor for 10 min at 25 °C. ATPase reaction started with the addition of 1 mM ATP. After 30 min of incubation at 25 °C, the assay was conducted following the manufacturer’s instructions. The ATPase enzymatic activity was analyzed using a 384-wells plate and the GloMax Discover Microplate Reader (Promega, MD, USA) and expressed as a percentage of inhibition calculated from the light units obtained.

### 3.4. In Vitro ADME

#### 3.4.1. HPLC/UV-MS Method

LC chromatographic analyses were performed by UV/LC-MS with an Agilent 1100 LC/MSD VL system (G1946C) (Agilent Technologies, Palo Alto, CA) equipped with a vacuum solvent degassing unit, a binary high-pressure gradient pump, an 1100 series UV detector, and a 1100 MSD model VL benchtop mass spectrometer. Chromatographic separations were obtained using a Phenomenex Kinetex C18-100 Å column (150 × 4.6 mm) with 5 µm particle size and gradient elution with a binary solution; (eluent A: H_2_O acidified with formic acid (FA) 0.1% *v*/*v*, eluent B: ACN/MeOH 1:1 *v*/*v*) at room temperature. The analysis started with 5% of B (from t = 0 to t = 1 min), then B was increased to 95% (from t = 1 to t = 10 min), then kept at 95% (from t = 10 to t = 15 min) and finally returned to 5% of eluent A in one minute. The instrument worked in positive mode and the UV detector operated at 254 nm.

#### 3.4.2. Aqueous Solubility

One mg of each compound was added with 1 mL of Mill-Q H_2_O. The samples were maintained under shaking at room temperature (RT) overnight. The suspensions were filtered using a 0.45 µm nylon filter (Acrodisc), and the amount of solubilized compound was determined with the HPLC-UV-MS method above reported. The quantification of the solubilized compound was created with the appropriate calibration curve realized with stock solutions in DMSO (0.1–100 µg/mL); the limit of detection (LOD) was quantified at 0.1 µg/mL.

#### 3.4.3. Parallel Artificial Membrane Permeability Assay (PAMPA)

In order to assess the apparent permeability of selected compounds, a stock solution in DMSO of each derivative was prepared at the final concentration of 1 mM. By diluting the stocks 1:1 *v*/*v* with phosphate buffer (PBS 25 mM, pH 7.4), donor solutions were made. To mimic the gastrointestinal (GI) phospholipidic bilayer, 10 µL of a 1% *w*/*v* dodecane solution of phosphatidylcholine (PC) was used to coat filters. The acceptor solution, made of 1:1 *v*/*v* DMSO/PBS, was added to each well (300 µL), while the donor solution (150 µL) was added to each well of the filter plate. The sandwich plates were assembled and incubated for 5 h at room temperature. At the time point, the plates were separated, and the amount of compound passed through the phospholipid bilayer was measured by UV/LC-MS. Finally, apparent permeability (P_app_) and membrane retention (MR%) were calculated as previously reported by us [21,22].

#### 3.4.4. Metabolic Stability Assay

A DMSO stock solution of tested compounds was incubated in triplicate in the presence of phosphate buffer (25 mM, pH 7.4), human liver microsomal protein (0.2 mg/mL), and in the presence of an NADPH regenerating system in MgCl_2_ (48 mM) at a final concentration of 50 µM. The metabolic reaction was conducted for 1 h under shaking at 37 °C and then stopped by adding 1.0 mL of cold acetonitrile (ACN). Centrifuging the reaction mixtures for 10 min at 5000 rpm, the supernatant was separated, dried under nitrogen flow, and finally resuspended in 100 µL of methanol (MeOH). The amount of parent drug and the metabolites were determined as previously described [23].

#### 3.4.5. Plasma Stability Assay

A DMSO stock solution of each compound was incubated in triplicate in the presence of human plasma and HEPES buffer (25 mM, 140 mM NaCl, pH 7.4) at the final concentration of 100 µM at 37 °C under shaking. At the selected time point of 0 and 24 h, 50 µL of the mixture was collected, treated with 1.0 mL of cold ACN, and centrifuged at 5000 rpm for 10 min. The supernatant was collected, and the amount of unmodified compound was quantified with the HLPC-UV/MS method above reported. Calculations of modified compounds were made using time zero as 100% of the unmodified compound.

## 4. Conclusions

In conclusion, a series of highly decorated triazines was designed and synthesized using the s-triazine privileged scaffold as the core structure. The novel derivatives were evaluated against SARS-CoV-2 resulting in the identification of compound **10a** as the hit of the series endowed with a micromolar activity. This compound showed also a very low cytotoxicity against Caco-2 cells, making it even more suitable for further optimizations. Preliminary study regarding the mechanism of action of **10a** showed the inhibition of the human DEAD-box RNA helicase DDX3X in the micromolar range as its parent derivatives **6** and **7** obtained in a previous study. Helicase inhibition, in fact, is often correlated with the reduction in the replication of several viruses at different steps such as the nuclear export of the newly synthesized genetic material of the pathogen [8,11,13,22].

In addition, in vitro ADME evaluations highlighted that all compounds tested (**6**, **7**, **10a–c**) were characterized by an excellent stability both in the presence of HLM and in plasma. While compound **6** and **7** showed a more lipophilic profile with a major affinity towards lipidic bilayer and lower aqueous solubility, compounds **10a–c** demonstrated a satisfactory passive permeability accompanied with low percentages of membrane retention and appreciable water solubility. These additional pharmacokinetic and enzymatic data further confirm the role of the hit compound of the series for **10a** and its potentiality as the starting point for future structure optimizations.

## Figures and Tables

**Table 1:** Antiviral activity, cytotoxicity, and selectivity index of the synthesized compounds in vitro in a cell-based model ^1^.

Entry	Compound	IC_50_ μM ^2^	CC_50_ μM ^3^	SI
1	**10a**	12.1	>400	>33.1
2	**10b**	NA	>200	-
3	**10c**	NA	184	-
4	**7**	NT	0.8	-
5	**13a**	NA	>200	-
6	**13b**	NA	>200	-
7	**6**	NT	3	-
8	**14a**	NT	70	-
9	**14b**	NA	22	-
10	Remdesivir	0.07 ± 0.04	94.9	1356

^1^ All experiments were conducted in Caco-2 cells in duplicate in three independent experiments; ^2^ IC_50_ half-maximal compound concentration inhibiting 50% of the SARS-CoV-2 replication; ^3^ CC_50_, half-maximal compound cytotoxic concentration, as determined by Cell-Titer Glo kit (Promega). SI, Selectivity Index (ratio between CC_50_ and IC_50_); NA—not active; NT—not tested, highly cytotoxic.

**Table 2:** Water solubility of compounds **6**, **7**, **10a–c**.

Cpd	µg/mL	LogS ^1^
**6**	<0.1	-
**7**	<0.1	-
**10a**	3.23	−5.07
**10b**	3.46	−5.09
**10c**	4.50	−4.93

^1^ Log of Solubility expressed as mol/L.

**Table 3:** In vitro PAMPA permeability studies of compounds **6**, **7**, **10a–c**.

Cpd	Papp ^a^	MR ^b^ (%)
**6**	4.74	28.7
**7**	4.58	15.5
**10a**	7.89	4.3
**10b**	9.70	9.8
**10c**	9.78	4.9

^a^ Apparent Permeability (Papp) reported in cm/s × 10^−6^. ^b^ Membrane retention %.

**Table 4:** Plasma stability studies after 24 h of compounds **6**, **7**, **10a–c**.

Plasma Stability % ± SD					
Time (h)	6	7	10a	10b	10c
0	100.00 ± 0.19	100.00 ± 0.28	100.00 ± 0.28	100.00 ± 0.24	100.00 ± 0.16
24	98.78 ± 4.05	99.56 ± 3.78	98.48 ± 4.02	98.41 ± 4.69	99.52 ± 3.45

## Data Availability

Not applicable.

## Supplementary Materials

The following supporting information can be downloaded at: https://www.mdpi.com/article/10.3390/molecules27248829/s1, synthetic procedures and full characterization of compounds synthesized (^1^H-NMR, ^13^C-NMR, MS); Table S1: Antiviral activity and cytotoxicity of the synthesized compounds in vitro in a cell-based model; Figure S1: Inhibition of the ATPase activity of the human helicase DDX3X.
