# Supplementary material for: Privileged Scaffold Decoration for the Identification of the First Trisubstituted Triazine with Anti-SARS-CoV-2 Activity
Source: Molecules. 2022 Dec 12;27(24):8829. doi: 10.3390/molecules27248829 (PMC9782877; doi:10.3390/molecules27248829)
Supplement: Supplementary file 1 [file molecules-27-08829-s001.zip › molecules-2036020-supplementary.pdf]

Supplementary Material

Privileged Scaffold Decoration for the Identification of the First Trisubstituted Triazine with Anti-SARS-CoV-2 Activity

Silvia Cesarini [1,†], Ilaria Vicenti [2,†], Federica Poggialini [3,†], Massimiliano Secchi [4,†], Federica Giammarino [2], Ilenia Varasi [2], Camilla Lodola [4], Maurizio Zazzi [2], Elena Dreassi [3], Giovanni Maga [4], Lorenzo Botta [1,*] and Raffaele Saladino [1]

1 Department of Biological and Ecological Sciences, Univeristy of Viterbo, Via S.C. De Lellis s.n.c., 01100 Viterbo, Italy
2 Department of Medical Biotechnologies, University of Siena, 53100 Siena, Italy
3 Department of Biotechnology, Chemistry, and Pharmacy (DBCF), University of Siena, 53100 Siena, Italy
4 Institute of Molecular Genetics, IGM CNR "Luigi Luca Cavalli-Sforza", Via Abbiategrasso 207, 27100 Pavia, Italy
* Correspondence: lorenzo.botta@unitus.it (L.B.)
† These authors contributed equally to this work.

1. Chemistry

Procedure for the synthesis of compounds 9a-b:

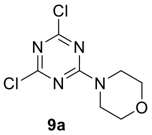

9a

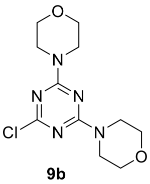

9b

To a stirred solution of cyanuric chloride **8** (200 mg, 1.1 mmol, 1 equiv.) in dimethoxyethane (DME, 5 mL) at -60 °C, morpholine (95 ◎L, 1.1 mmol, 1 equiv.) was added dropwise. The reaction mixture was vigorously stirred for 3 h and then warmed to 25 °C. The resulting mixture was diluted with ethyl acetate (EtOAc, 20 mL) and washed successively with 3N HCl (20 mL), H_2O (20 mL), and brine (20 mL). The organic phase was dried over anhydrous Na_2SO_4 and then evaporated to dryness. The resulting white residue was purified by column chromatography using a mixture of petroleum ether (PET)/diethyl ether (Et_2O) (4:1) to give 141 mg (0.6 mmol) of **9a** and 47 mg (0.165 mmol) of **9b** with 55% and 15% of yield respectively.

Monosubstituted triazine 9a:

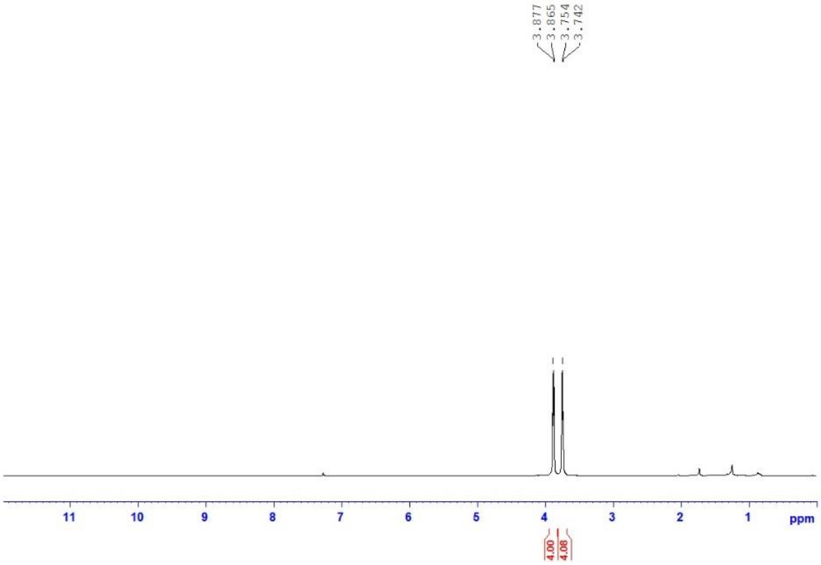

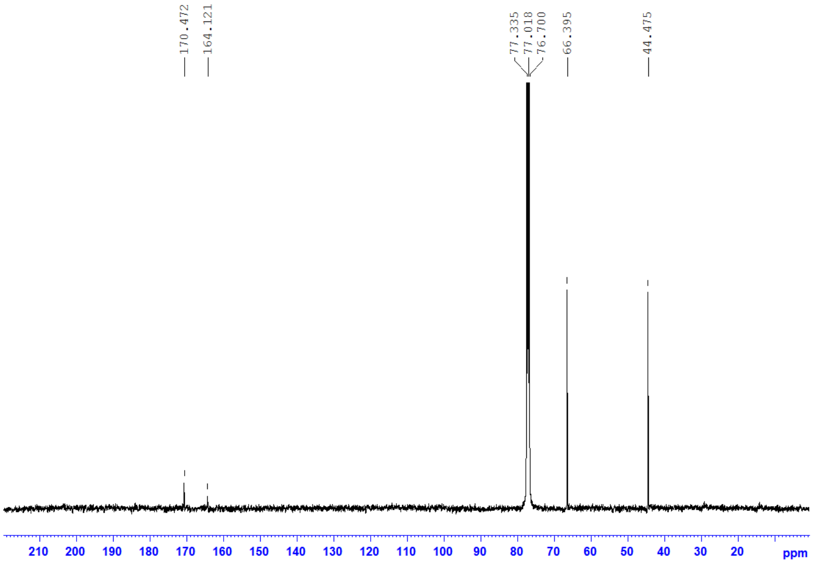

White powder; Yield = 55%; R_f = 0.30 (PET/Et_2O, 4:1);

^{1}H NMR (400 MHz, $CDCl_3$): δ=3.87 (d, J=4.8 Hz, 4H); 3.75 (d, J= 4.8 Hz, 4H); ^{13}C NMR (100 MHz, $CDCl_3$): δ=170.5, 164.1, 66.4, 44.4 ppm. MS m/z for $C_7H_8Cl_2N_4O$, (ESI$^+$) m/z: 235.07 $[M+H]^+$.

Disubstituted triazine 9b:

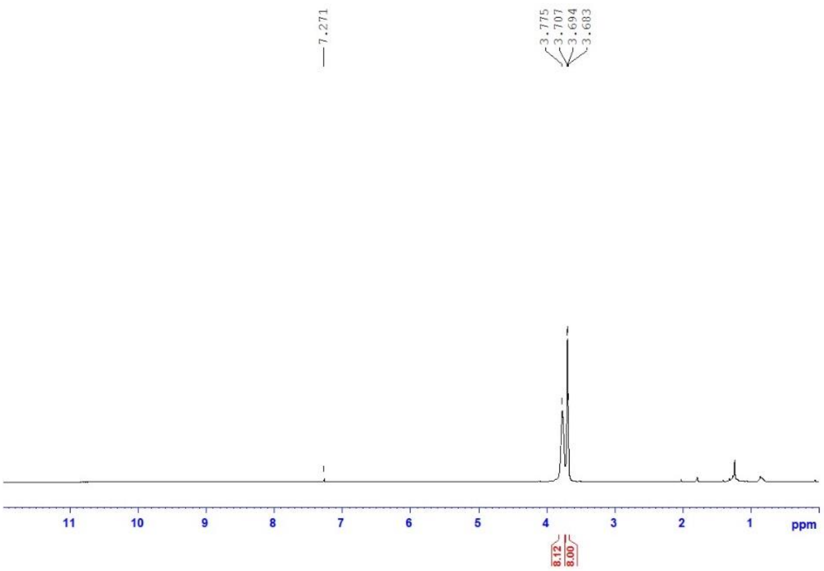

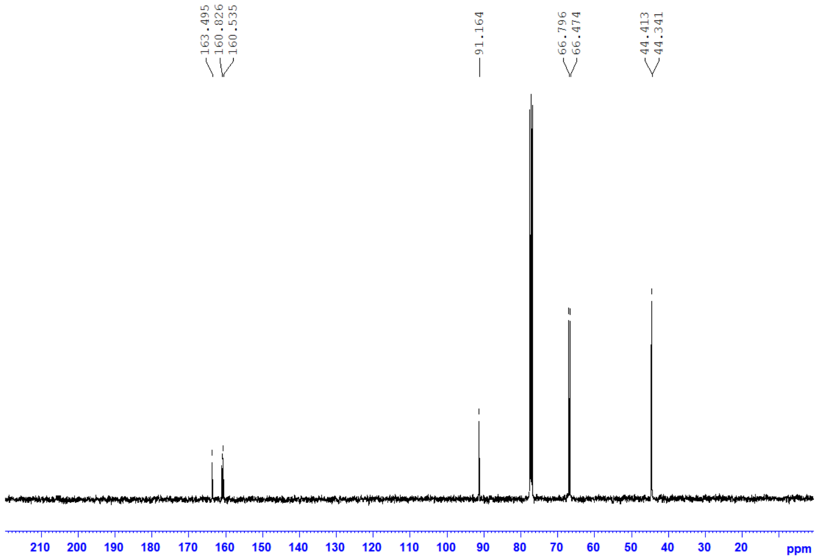

White powder (15%); R_f = 0.25 (PET/Et_2O, 4:1);

^{1}H NMR (400 MHz, $CDCl_3$): δ=3.78 (bs, 8 H); 3.69 (t, J=4.4 Hz, 8 H); ^{13}C NMR (100 MHz, $CDCl_3$): δ=163.5, 160.8, 160.5, 91.2, 66.8, 66.5, 44.4, 44.3 ppm. MS m/z for $C_{11}H_{16}ClN_5O_2$, (ESI$^+$) m/z: 286.0 $[M+H]^+$.

Procedure for the direct synthesis of compound 9b:

Cyanuric chloride **8** (200mg, 1.1 mmol, 1 equiv.), *N,N*-Diisopropylethylamine (DIPEA) (421 ◎L, 2.42 mmol, 2.2 equiv.), and morpholine (209 ◎L, 2.42 mmol, 2.2 equiv.) were dissolved in ethanol (10 mL). The reaction mixture was stirred at 25 °C for 18 h. After that the volatiles were removed under reduced pressure using a rotary evaporator. The resulting mixture was dissolved in dichloromethane (CH_2Cl_2, 10 mL), and washed twice with s.s.$NaHSO_4$ (2x10). The organic layer was dried over anhydrous Na_2SO_4, filtered and concentrated under reducing pressure.

The resulting residue was purified by crystallization from MeOH/DCM to give **9b** (125 mg, 0.44 mmol, 40% of yield).

Procedure for the synthesis of 9b from derivative 9a:

To a solution of the monosubstituted product **9a** (125 mg, 0.53 mmol, 1 equiv.) in CH_2Cl_2 (5 mL), morpholine (92 ◎L, 1.06 mmol, 2 equiv.) was added dropwise. The reaction was stirred at reflux for 18 h. After cooling to 25 °C, the mixture was diluted with CH_2Cl_2 (10 mL), and washed with 3N HCl (15 mL), H_2O (15 mL), and brine (15 mL). The organic phase was then dried over anhydrous Na_2SO_4 and evaporated to dryness. The resulting residue was purified by crystallization from CH_2Cl_2 to give compound **9b** (140 mg, 0.49 mmol, 92% of yield).

General Procedure for the synthesis of triazine derivatives 10 a-c:

To a solution of intermediate **9b** (100 mg, 0.35 mmol, 1 equiv.) in CH_2Cl_2 (3 mL), hydrazine hydrate (45 ◎L, 1.4 mmol, 4 equiv.) was added and the resulting mixture was held at reflux for 12 h. After cooling to 25 °C, the mixture was diluted in CH_2Cl_2 (10 mL) washed with H_2O (10 mL) and brine (10 mL). The organic phase was dried over anhydrous Na_2SO_4 and evaporated to dryness. The obtained residue was used for the next step without further purification.

The residue was dissolved in toluene (3 mL) and reacted with the appropriate aldehyde (0.7 mmol, 2 equiv.). The reaction mixture was held at reflux for 3 h using a Dean–Stark apparatus for azeotropical removal of H_2O and then evaporated to dryness. The resulting residue was dissolved in a minimum amount of CH_2Cl_2. Upon addition of PET, the desired final compounds **10 a-c** precipitated and were collected by filtration with 50, 45 and 25 % of yield.

Highly decorated triazine 10a:

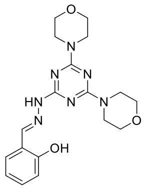

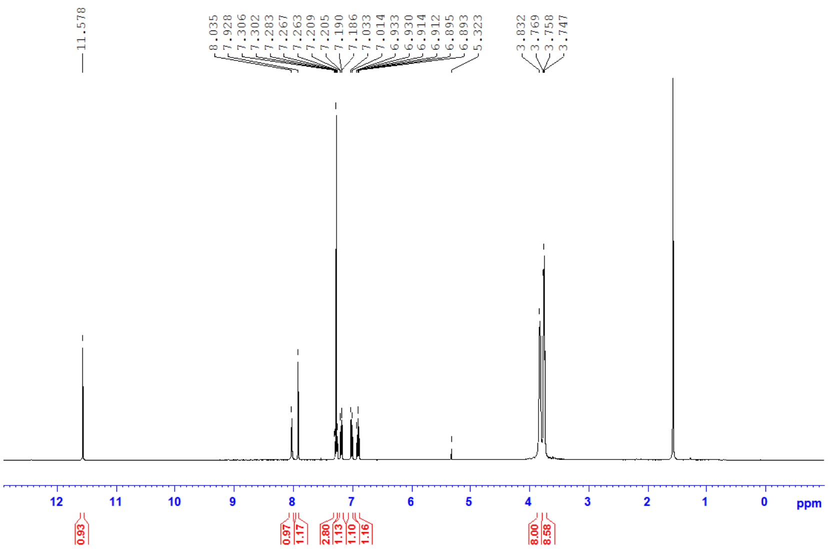

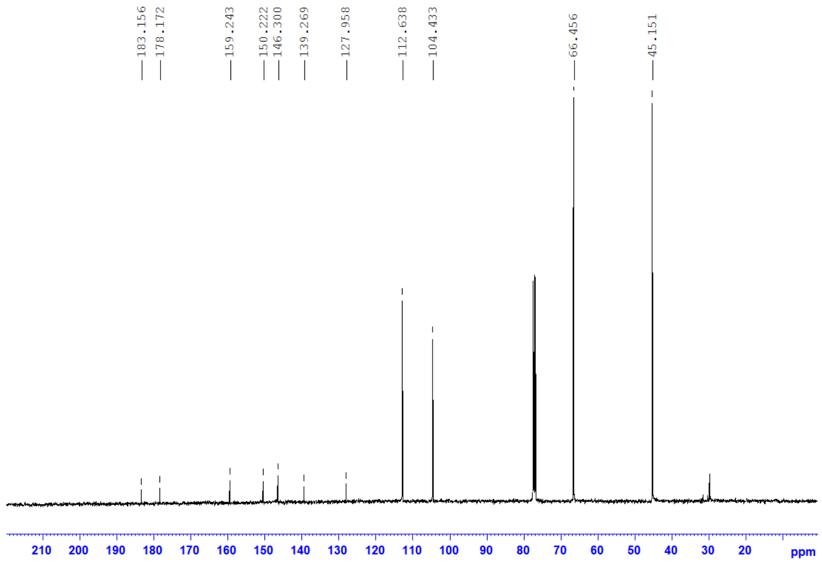

White powder (67 mg, 0.175 mmol, 50%); $R_f = 0.31$; (PET/EtOAc, 2:1);

^{1}H NMR (400 MHz, $CDCl_3$): δ=11.58 (s, 1H), 8.04 (s, 1H), 7.93 (s, 1H), 7.30-7.26 (m, 1H), 7.21-7.19 (m, 1H), 7.03-7.01 (d, J= 7.6 Hz, 1H), 6.93-6.89 (m, 1H) 3.83 (s, 8H), 3.76 (t, J= 4.4 Hz, 8H); ^{13}C NMR (100 MHz, $CDCl_3$): 183.2, 178.2, 159.2, 150.2, 146.3, 139.3, 128.0, 112.6, 104.4, 66.5, 45.2. ppm. MS m/z for $C_{18}H_{23}N_7O_3$, (ESI$^+$) m/z: 386.2 $[M+H]^+$.

Highly decorated triazine 10b:

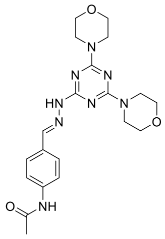

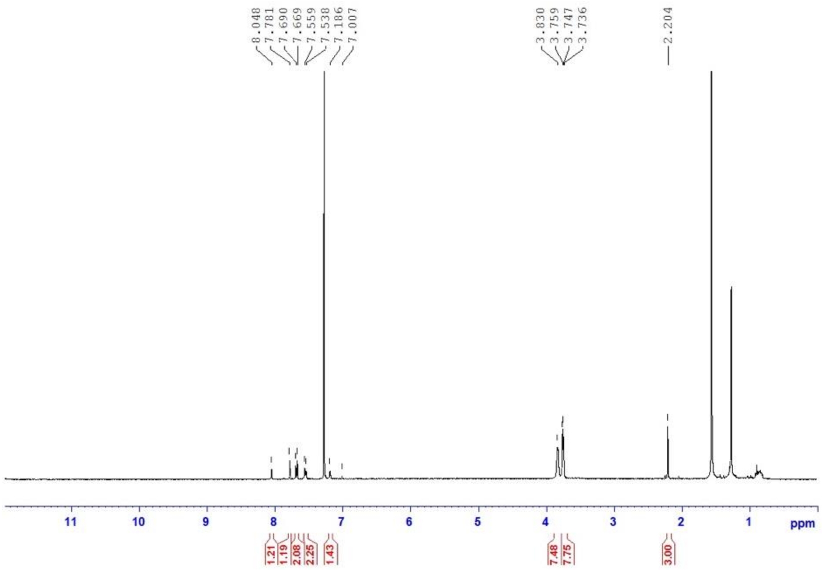

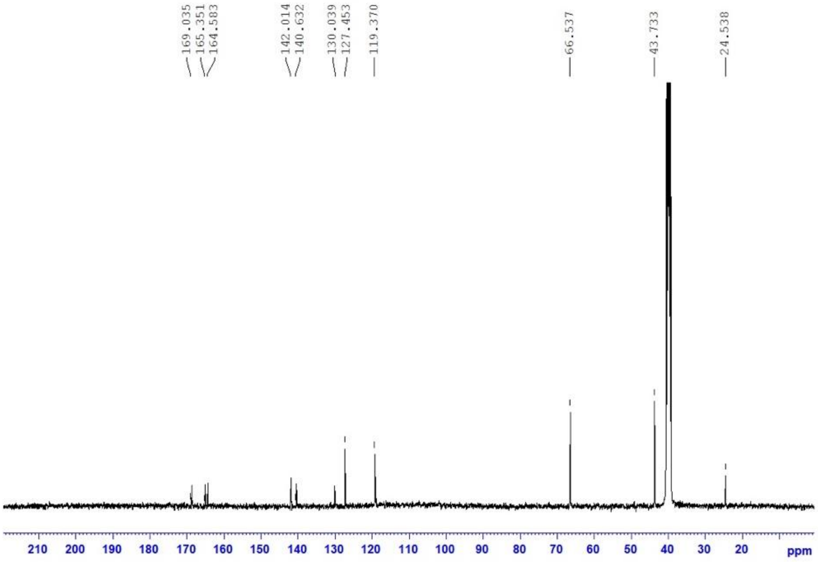

White powder (37 mg, 0.088 mmol, 25%), R_f = 0.18 (HEX/EtOAc, 2:1);

^{1}H NMR (400 MHz, $CDCl_3$): δ=8.05 (s, 1H), 7.78 (s, 1H), 7.68 (d, *J*=8.4 Hz, 2H), 7.55 (d, *J*=8.4 Hz, 2H),7.19 (s, 1H), 3.83 (bs, 8H), 3.75 (t, *J*= 4.6 Hz, 8H), 2.20 (s, 3H); ^{13}C NMR (100 MHz, $CDCl_3$): δ= 169.0, 165.4, 164.6, 142.0, 140.6, 130.0, 127.5, 119.4, 66.5, 43.7, 24.5 ppm. MS *m/z* for $C_{20}H_{26}N_8O_3$, (ESI$^+$) *m/z*: 427.1 [M+H]$^+$.

Highly decorated triazine 10c:

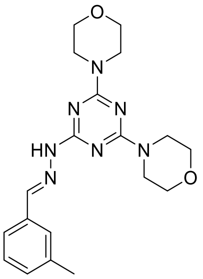

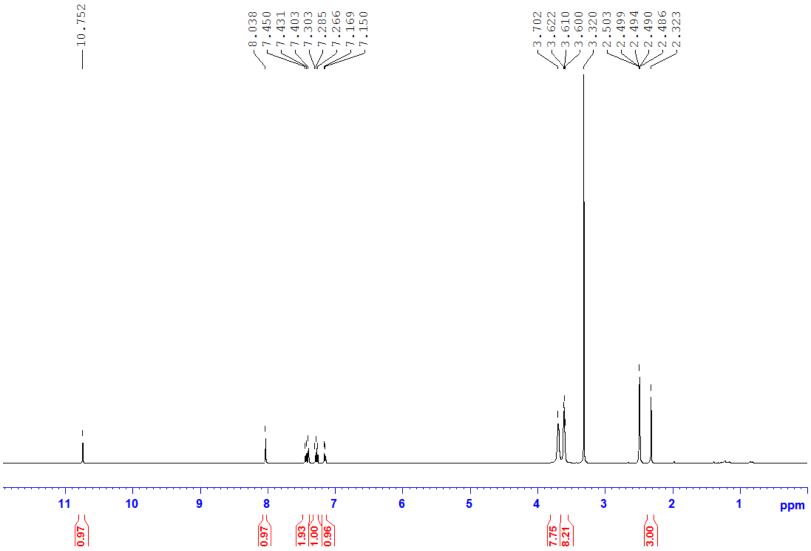

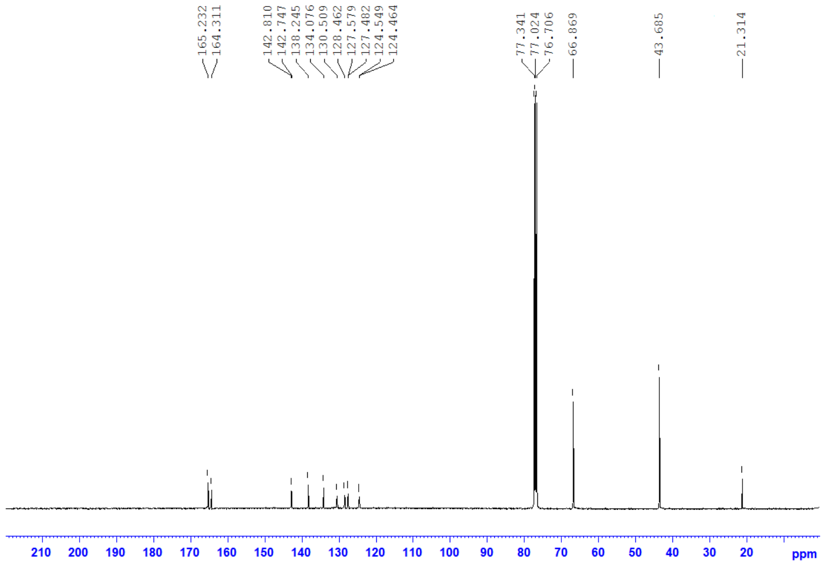

White powder (61 mg, 0.16 mmol, 45%); R_f = 0.21 (HEX/EtOAc, 1:1);

^{1}H NMR (400 MHz, DMSO-d_6) δ 10.75 (s, 1H), 8.03 (s, 1H) 7.43 (t, *J*= 9.4 Hz, 2H), 7.28 (t, *J*=7.6 Hz, 1H), 7.16 (d, *J*=7.6 Hz, 1H), 3.70 (s, 8H), 3.61 (t, *J*= 4.8 Hz, 8H), 2.32 (s, 3H). ^{13}C NMR (100 MHz, $CDCl_3$) δ 165.2, 164.3, 142.8, 142.7, 138.2, 134.1, 130.5, 128.4, 127.6, 127.5, 124.5, 124.4, 66.9, 43.7, 21.3. MS *m/z* for $C_{19}H_{25}N_7O_2$, (ESI$^+$) *m/z*: 384.23 $[M+H]^+$.

Procedure for the synthesis compounds 12a-b:

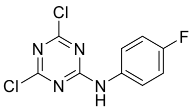

12a

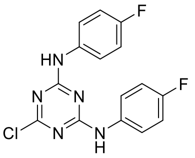

12b

To a stirred solution of cyanuric chloride **8** (500 mg, 2.72 mmol, 1 equiv.) in dimethoxyethane (10 mL) at -60 °C, 4-fluoroaniline (257 ◎L, 2.72 mmol, 1 equiv.) was added dropwise. The reaction mixture was vigorously stirred for 3 h, then warmed to 25 °C. The resulting mixture was diluted with EtOAc (10 mL), washed successively with 3N HCl (20 mL), H_2O (20 mL), and brine (20 mL). The organic phase was dried over anhydrous Na_2SO_4 and then evaporated to dryness. The resulting white residue was purified by column chromatography using a mixture of PET/Et_2O (4:1) to give 332 mg (1.28 mmol) of **12a** and 91 mg (0.27 mmol) of **12b** with 47% and 10% of yield respectively.

Monosubstituted triazine 12a:

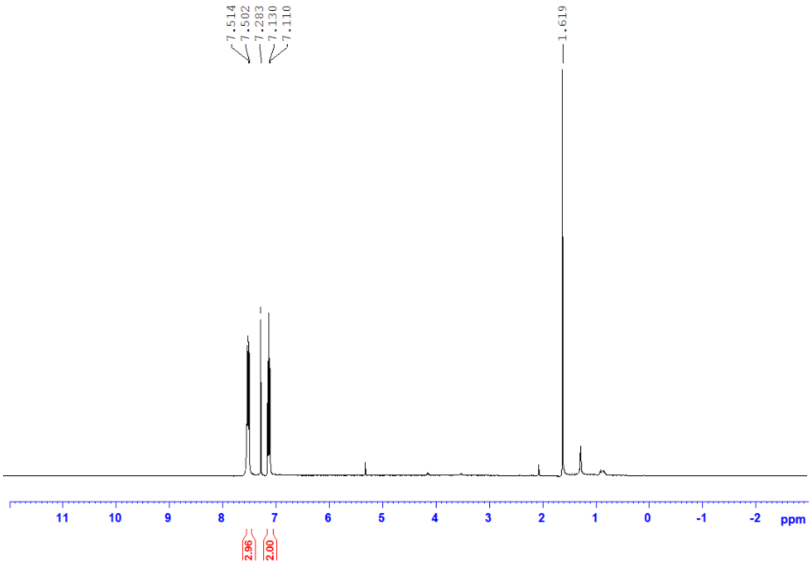

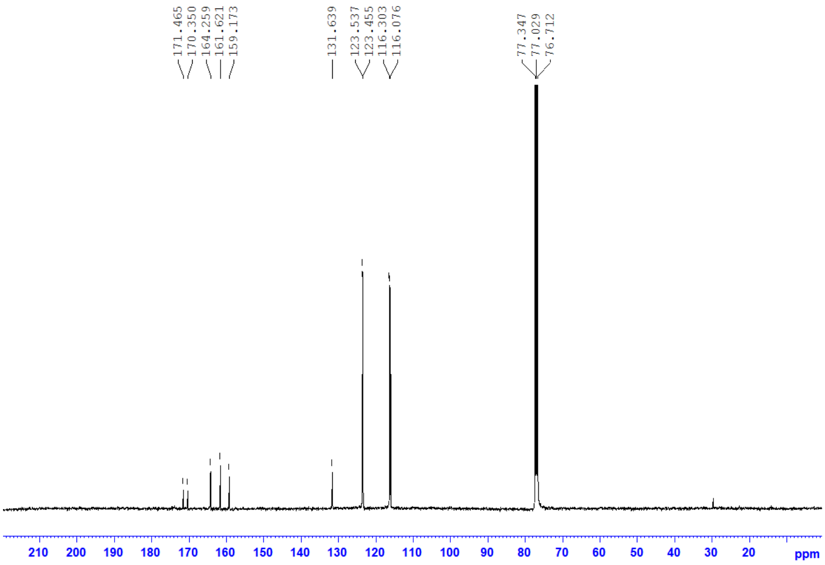

White solid (47 %); R_f: 0.25 (HEX/EtOAc, 2:1);

^{1}H NMR (400 MHz, $CDCl_3$) δ 7.51 (d, *J*=4.8 Hz, 2H), 7.12 (d, *J*=8 Hz, 2H). ^{13}C NMR (100 MHz, $CDCl_3$) δ 171.5, 170.3, 164.3, 161.6, 159.2, 131.6, 123.5, 123.4, 116.3, 116.1 ppm. MS *m*/*z* for $C_9H_5Cl_2FN_4$, (ESI$^-$) *m*/*z*: 257.0 [M+H].

Disubstituted triazine 12b:

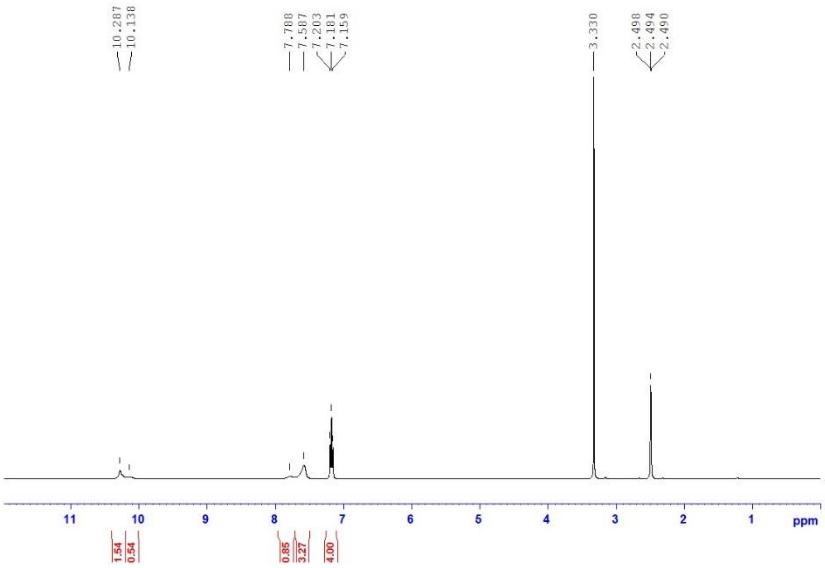

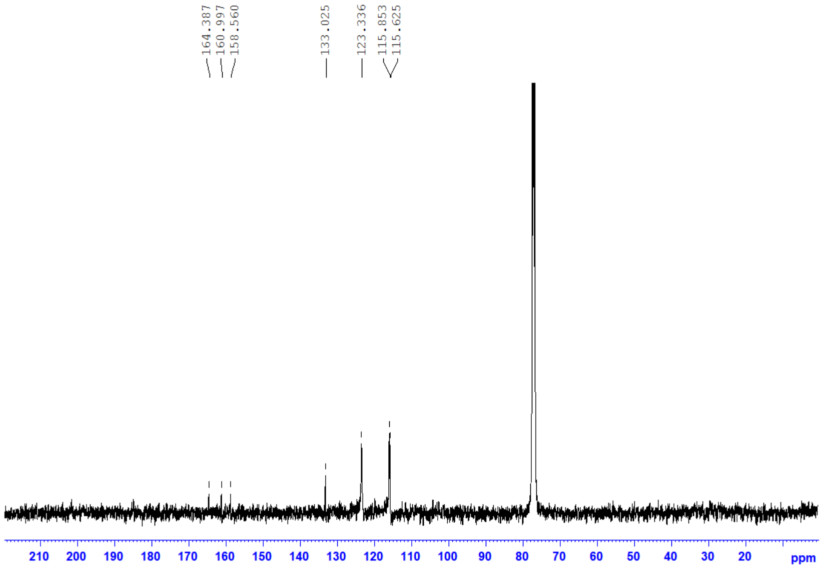

White solid (10 %); R_f: 0.17 (HEX/EtOAc, 2:1);

^{1}H NMR (400 MHz, DMSO-*d6*) δ 10.29 (bs, 1H), 7.79 (bs, 1H), 7.59 (bs, 4H), 7.18 (t, *J*= 8.8 Hz, 4H). ^{13}C NMR (100 MHz, $CDCl_3$) δ 164.4, 160.9, 158.6, 133.0, 123.3, 115.8, 115.6. MS *m/z* for $C_{15}H_{10}ClF_2N_5$, (ESI$^-$) *m/z*: 332.0 $[M+H]^-$.

Procedure for the direct synthesis of compound 12b:

Cyanuric chloride **8** (100 mg, 0.54 mmol, 1 equiv.), DIPEA (207 µL, 1.19 mmol, 2.2 equiv.), and 4-fluoroaniline (113 µL, 1.19 mmol, 2.2 equiv.) were dissolved in ethanol (4 mL). The reaction mixture was stirred at 25 °C for 18 h. After that the volatiles were removed under reduce pressure using a rotary evaporator. The resulting mixture was dissolved in CH_2Cl_2 (10 mL), and washed twice with s.s.$NaHSO_4$ (2x10). The organic layer was dried over anhydrous Na_2SO_4, filtered and concentrated under reducing pressure.

The resulting residue was purified by crystallization from MeOH/DCM to give 117 mg of **12b** (0.35 mmol, 65% of yield).

Procedure for the synthesis of 12b from derivative 12a:

To a solution of the monosubstituted product **12a** (150 mg, 0.58 mmol, 1 equiv.) in CH_2Cl_2 (3 mL), DIPEA (303 ◎L, 1.74 mmol, 3 equiv.) and 4-fluoroaniline (82 ◎L, 0.87 mmol, 1.5 equiv.) were added dropwise. The reaction was stirred at reflux for 18 h and then then warmed to 25 °C. The resulting mixture was diluted with CH_2Cl_2 (10 mL) and washed with 1N HCl (10 mL), H_2O (10mL), and brine (10 mL). The organic phase was then dried over anhydrous Na_2SO_4 and evaporated to dryness. The resulting residue was purified by crystallization from CH_2Cl_2 to give 143 mg (0.43 mmol) of compound **12b** with 74% of yield.

General procedure for the synthesis of triazine derivatives 7, 13a and 13b:

To a solution of intermediate **12b** (163 mg, 0.49 mmol, 1 equiv.) in CH_2Cl_2 (5 mL), hydrazine hydrate (62 ◎L, 1.96 mmol, 4 equiv.) was added and the resulting mixture was held at reflux for 12 h. After cooling to 25 °C, the mixture was diluted in CH_2Cl_2 (10 mL) washed with H_2O (20 mL) and brine (20 mL). The organic phase was dried over anhydrous Na_2SO_4 and evaporated to dryness. The obtained residue was used for the next step without further purification.

The residue was dissolved in MeOH (5 mL) and reacted with the appropriate aldehyde (0.49 mmol, 1 equiv.). Two drops of glacial acetic acid were then added. After the mixture was allowed to stand at 25 °C for 18 hours, the precipitated product was filtered, washed with MeOH and dried under reduce pressure to obtain compounds **7**, **13a** and **13b** with 50, 53 and 55 % of yield respectively.

Highly decorated triazine 7

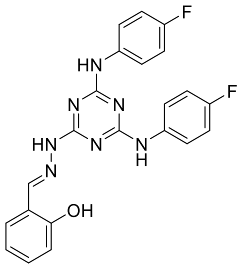

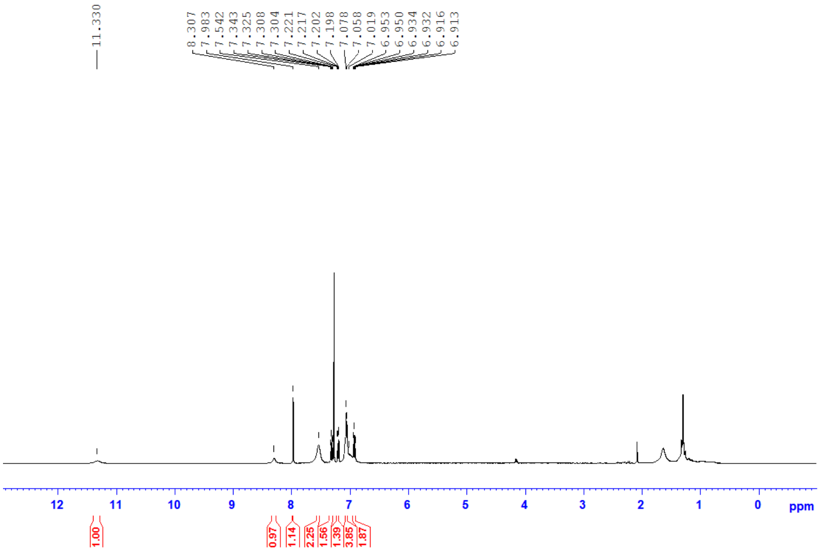

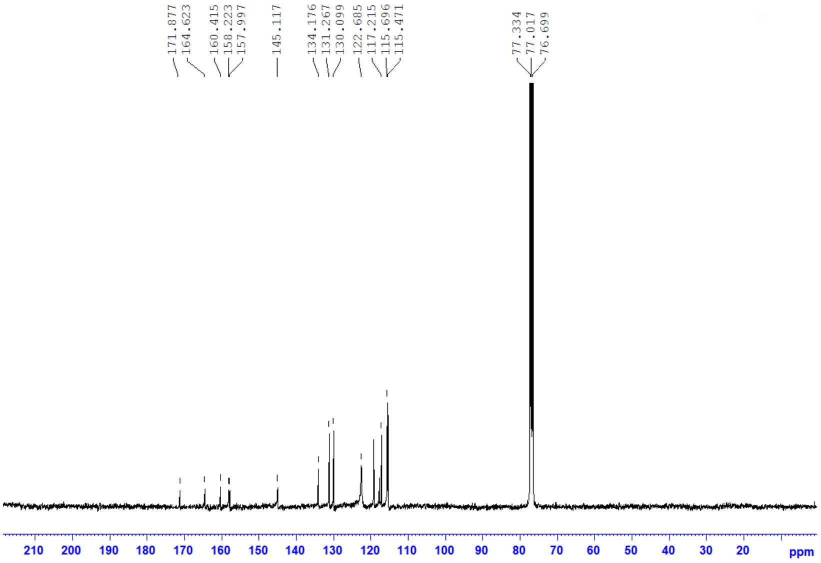

White solid (106 mg, 0.245 mmol, 50%); R_f: 0.23 (HEX/EtOAc, 2:1)

^{1}H NMR (400 MHz, $CDCl_3$) δ 11.33 (bs, 1H), 8.31 (bs, 1H), 7.98 (s,1H), 7.54 (bs,3H), 7.34-7.30 (m, 2H), 7.22-7.19 (m,1H), 7.07-6.95 (m, 4H), 6.93-6.91 (m, 2H). ^{13}C NMR (100 MHz, $CDCl_3$) δ 171.9, 164.6, 160.4, 158.2, 158.0, 145.1, 134.2, 131.3, 130.1, 122.7, 117.2, 115.7, 115.5 ppm. MS *m/z* for $C_{22}H_{17}F_2N_7O$, (ESI$^+$) *m/z*: 434.15 $[M+H]^+$.

Highly decorated triazine 13a:

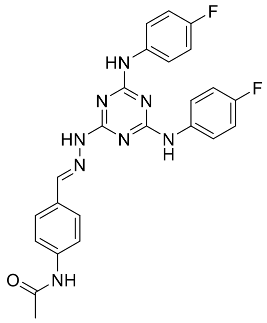

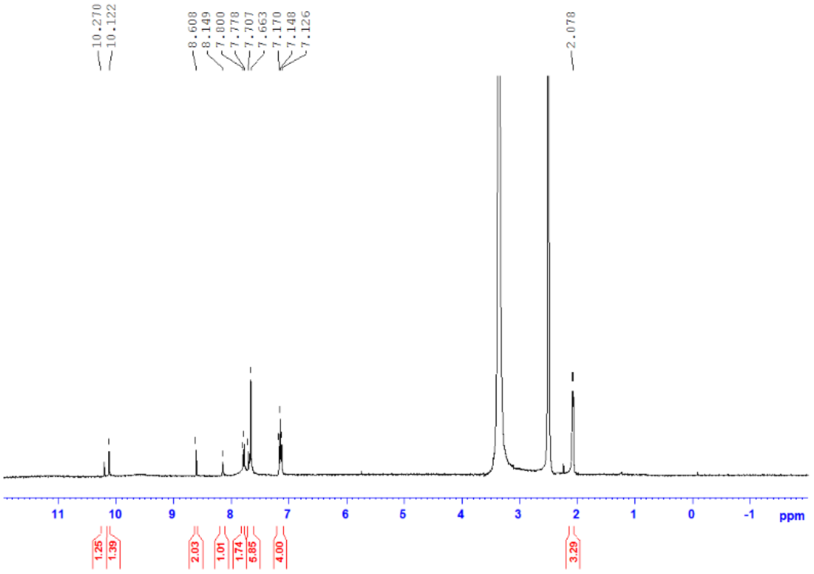

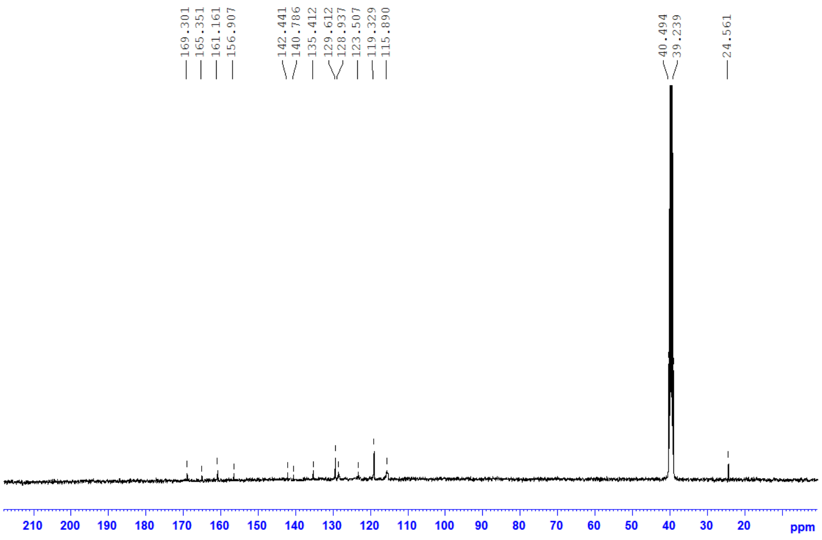

White solid (123 mg, 0.26 mmol, 53%); R_f: 0.27 (HEX/EtOAc, 2:1);

^{1}H NMR (400 MHz, DMSO-d_6) δ 10.27 (s, 1H), 10.12 (s, 1H), 8.60 (s, 2H), 8.15 (s, 1H), 7.79 (d, *J*= 8.8 Hz, 2H), 7.71-7.66 (m, 6H), 7.15 (t, *J*= 8.8 Hz, 4H), 2.08 (s, 3H). ^{13}C NMR (100 MHz, DMSO-d_6) δ 169.3, 165.4, 161.2, 156.9, 142.4, 140.8, 135.4, 129.6, 128.9, 123.5, 119.3, 115.9, 24.6 ppm. MS *m/z* for $C_{22}H_{17}F_2N_7O$, (ESI$^+$) *m/z*: 475.1 [M+H]$^+$; (ESI$^-$) *m/z*: 473.1 [M-H]$^-$, 509.2 [M+Cl]$^-$.

Highly decorated triazine 13b:

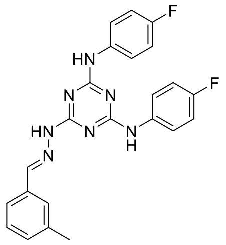

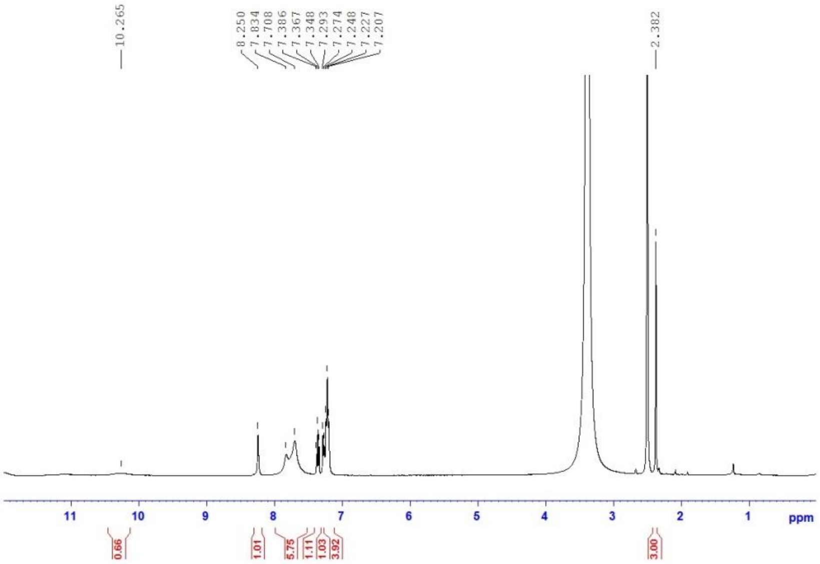

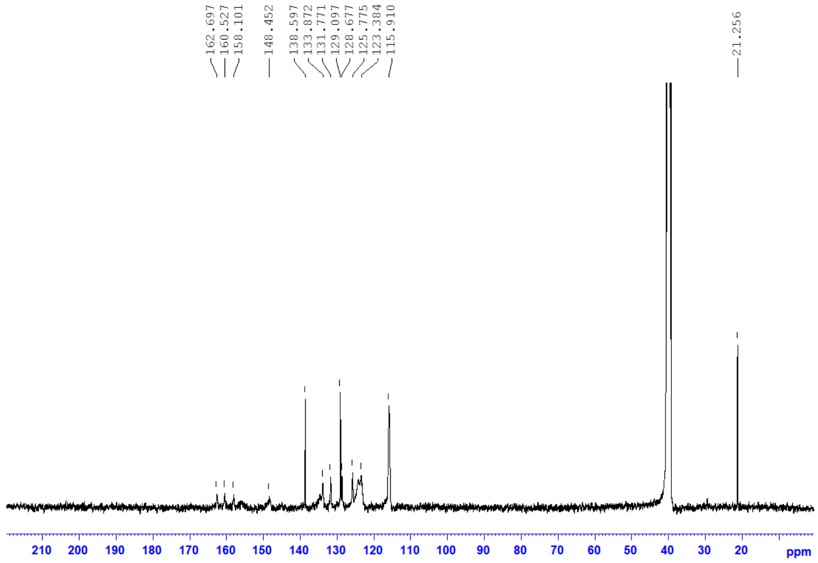

White solid (116 mg, 0.27 mmol, 55%); R_f: 0.21 (HEX/EtOAc, 2:1)

^{1}H NMR (400 MHz, DMSO-d_6) δ 10.27 (bs, 1H), 8.25 (s, 1H), 8.05-7.46 (m, 6H), 7.37 (t, *J*= 7.6 Hz, 1H), 7.28 (d, *J*= 7.6 Hz, 1H), 7.23 (t, *J*= 8.4 Hz, 4H), 2.38 (s, 3H). ^{13}C NMR (100 MHz, DMSO-d_6) 162.7, 160.5, 158.1, 148.5, 138.6, 133.9, 131.8, 129.1, 128.7, 125.8, 123.4, 115.9, 21.3 ppm. MS *m/z* for $C_{23}H_{19}F_2N_7$, (ESI$^+$) *m/z*: 432.1 [M+H]$^+$; (ESI$^-$) *m/z*: 430.1 [M-H]$^-$.

Procedure for the synthesis of a disubstituted triazine derivative 15:

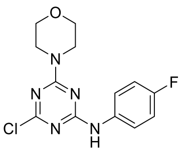

To a solution of the monosubstituted product **12a** (95 mg, 0.37 mmol, 1 equiv.) in CH_2Cl_2 (4 mL), morpholine (64 ◎L, 0.74 mmol, 2 equiv.) was added dropwise. The resulting mixture was stirred for 5h at 25 °C and then diluted with CH_2Cl_2 (10 mL), washed with 3N HCl (10 mL), H_2O (10mL), and brine (10 mL). The organic phase was then dried over anhydrous Na_2SO_4 and evaporated to dryness. The obtained disubstituted product was dissolved in a minimum amount of CH_2Cl_2. Upon addition of PET, compound **15** precipitated and was collected by filtration.

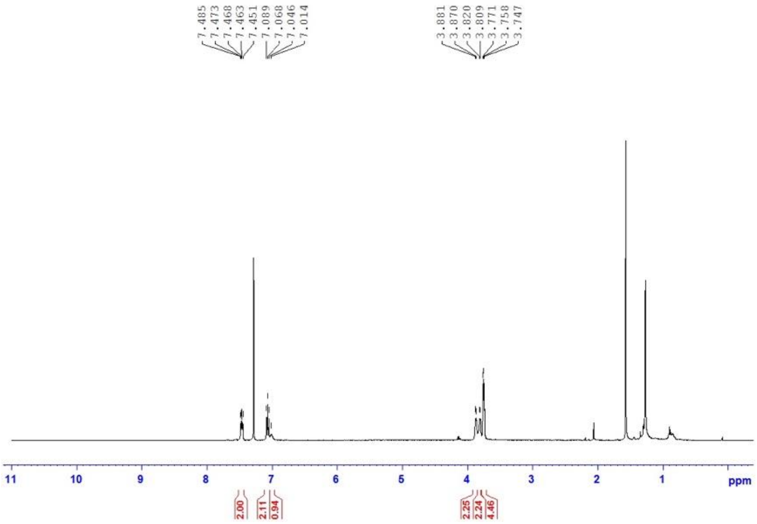

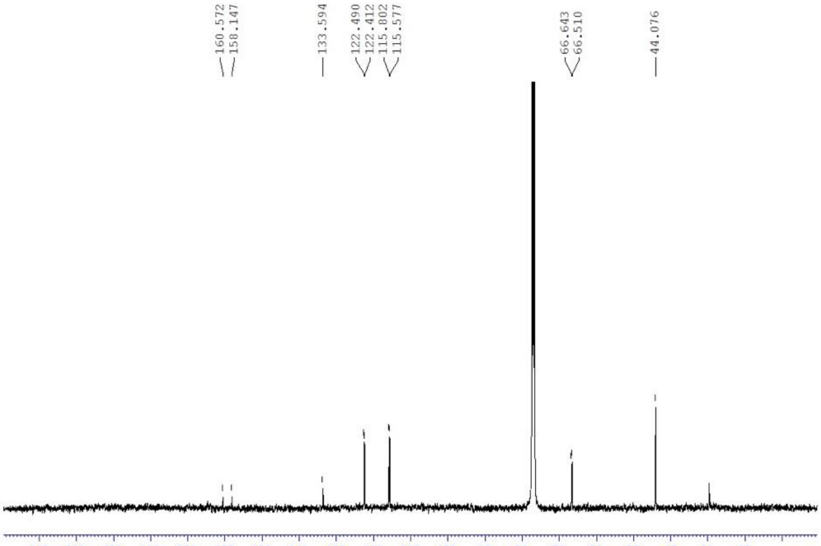

White solid (108 mg, 0.35 mmol, 94 %); R_f: 0.28 (HEX/EtOAc, 3:1);

^{1}H NMR (400 MHz, $CDCl_3$) δ 7.48-7.45 (m, 2H), 7.07 (t, *J*=8.4 Hz, 2H), 7.01 (bs, 1H), 3.91-3.85 (m, 2H), 3.85-3.79 (m, 2H), 3.76 (d, *J*=4.4 Hz, 4H). ^{13}C NMR (100 MHz, $CDCl_3$) δ 160.6, 158.1, 133.6, 122.5, 122.4, 115.8, 115.6, 66.6, 66.5, 44.1 ppm. MS *m/z* for $C_{13}H_{13}ClFN_5O$, (ESI$^+$) *m/z*: 310.1 [M+H]$^+$; (ESI$^-$) *m/z*: 308.0 [M+H]$^-$.

General procedure for the synthesis of triazine derivatives 6, 14a and 14b:

To a solution of intermediate **15** (100 mg, 0.32 mmol, 1 equiv.) in CH_2Cl_2 (4 mL), hydrazine hydrate (41 ◎L, 1.28 mmol, 4 equiv.) was added and the resulting mixture was held at reflux for 12 h. After cooling to 25 °C, the mixture was diluted in CH_2Cl_2 (10 mL) washed with H_2O (20 mL) and brine (20 mL). The organic phase was dried over anhydrous Na_2SO_4 and evaporated to dryness. The obtained residue was used for the next step without further purification.

The residue was dissolved in MeOH (5 mL) and reacted with the appropriate aldehyde (0.32 mmol, 1 equiv.). Two drops of glacial acetic acid were then added. After the mixture was allowed to stand at 25 °C for 18 hours, the precipitated product was filtered, washed with MeOH and dried under reduce pressure to obtain compounds **6**, **14a** and **14b** with 45, 52 and 46% of yield respectively.

Highly decorated triazine 6:

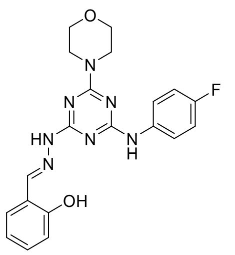

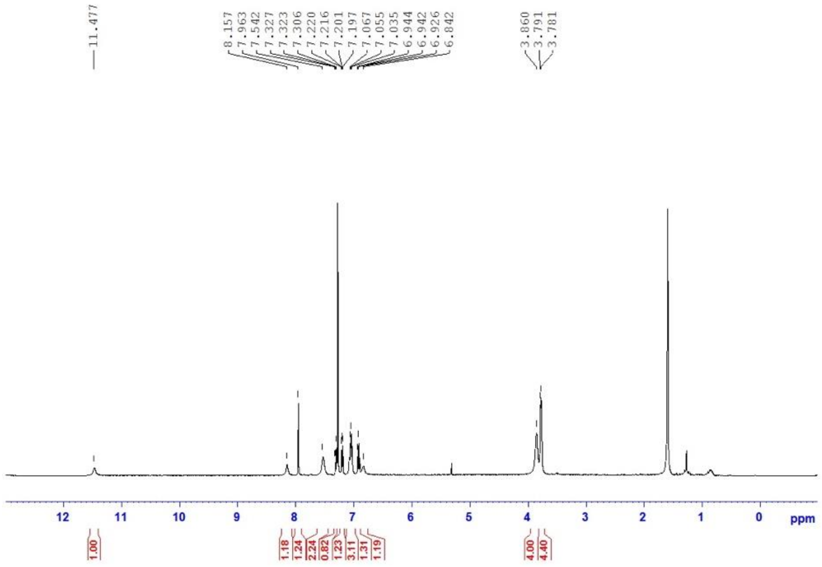

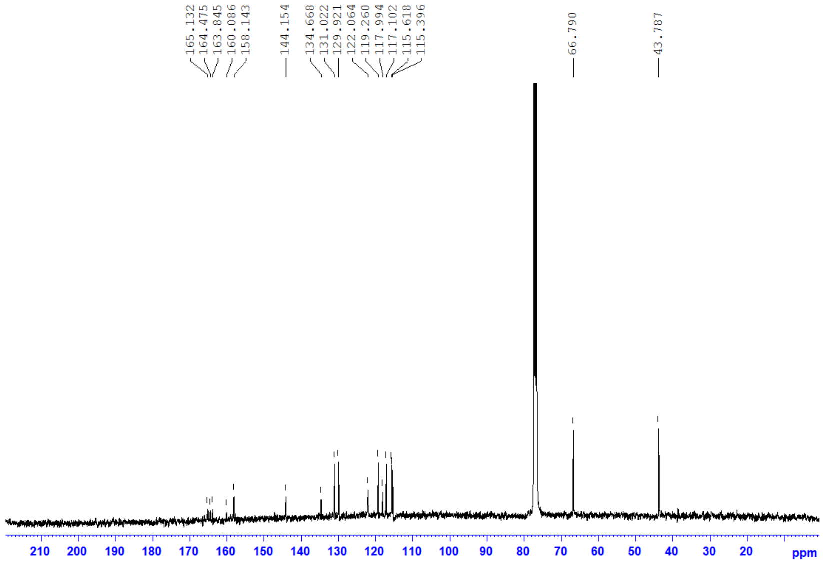

White powder (59 mg, 0.144 mmol, 45%); R_f = 0.19 (HEX/EtOAc, 3:1);

^{1}H-NMR ($CDCl_3$, 400 MHz) δ=11.48 (s, 1H), 8.16 (bs, 1H), 7.96 (s, 1H), 7.54 (bs, 2H), 7.33 (d, *J*=7.2 Hz, 1H), 7.22 (d, *J*=7.6 Hz, 1H), 7.20 (d, *J*=4.6 Hz, 1H), 7.07-7.01 (m, 3H), 6.94-6.92 (m, 1H), 6.84 (bs, 1H), 3.86 (bs, 4H), 3.79 (d, *J*=4.0 Hz, 4H) ppm. ^{13}C-NMR ($CDCl_3$, 100 MHz) δ=165.1, 164.5, 163.8, 160.09, 158.1, 144.2, 134.7, 131.0, 129.9, 122.1, 119.3, 117.9, 117.1, 115.6, 115.4, 66.8, 43.8, ppm. MS *m/z* for $C_{20}H_{20}ClFN_7O_2$, (ESI$^+$) *m/z*: 410.2 $[M+H]^+$.

Highly decorated triazine 14a:

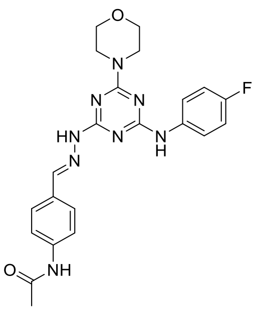

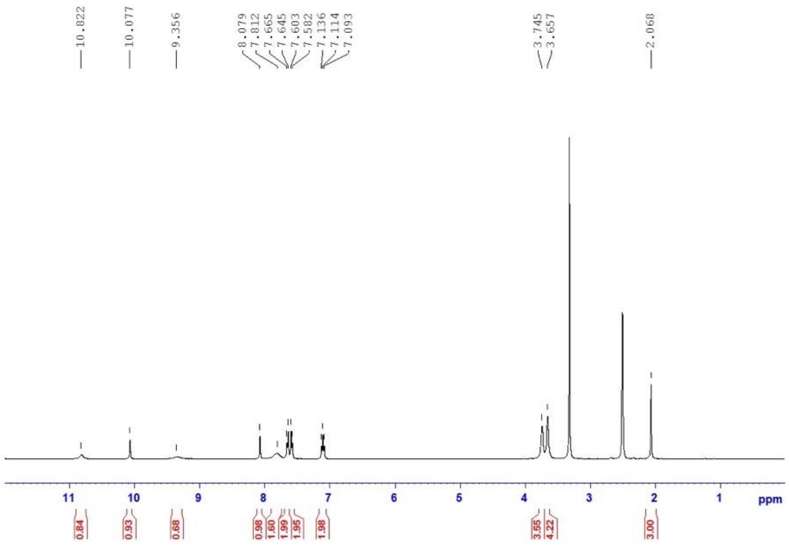

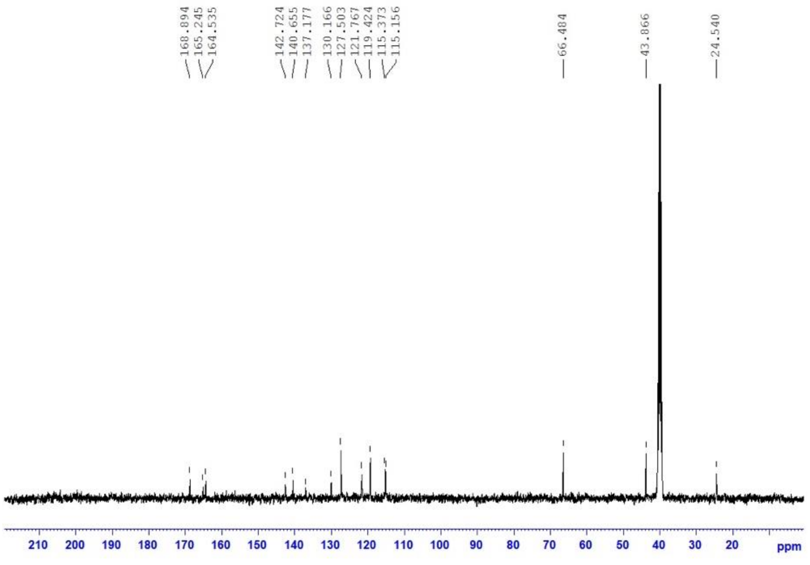

White solid (75 mg, 0.166 mmol, 52%); R_f: 0.20 (HEX/EtOAc, 3:1)

^{1}H NMR (400 MHz, $CDCl_3$) δ 10.82 (bs, 1H); 10.08 (s, 1H), 9.36 (bs, 1H), 8.08 (s, 1H), 7.82 (bs, 2H), 7.66 (d, *J*=8.0 Hz, 2H), 7.59 (t, *J*=8.4 Hz, 2H), 7.11 (t, *J*= 8.8 Hz, 3H), 3.75 (bs, 4H), 3.66 (bs, 4H), 2.07 (s, 3H). ^{13}C NMR (100 MHz, $CDCl_3$) δ 168.9, 165.2, 164.5, 142.7, 140.7, 137.2, 130.2, 127.5, 121.8, 119.4, 115.4, 115.2, 66.5, 43.9, 24.5 ppm. MS *m/z* for $C_{22}H_{23}FN_8O_2$, (ESI$^+$) *m/z*: 451.2 $[M+H]^+$; (ESI$^-$) *m/z*: 449.1$[M-H]^-$, 485.1 $[M+Cl]^-$.

Highly decorated triazine 14b:

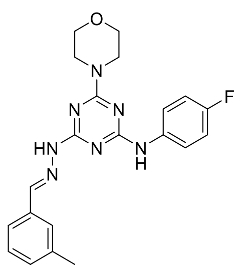

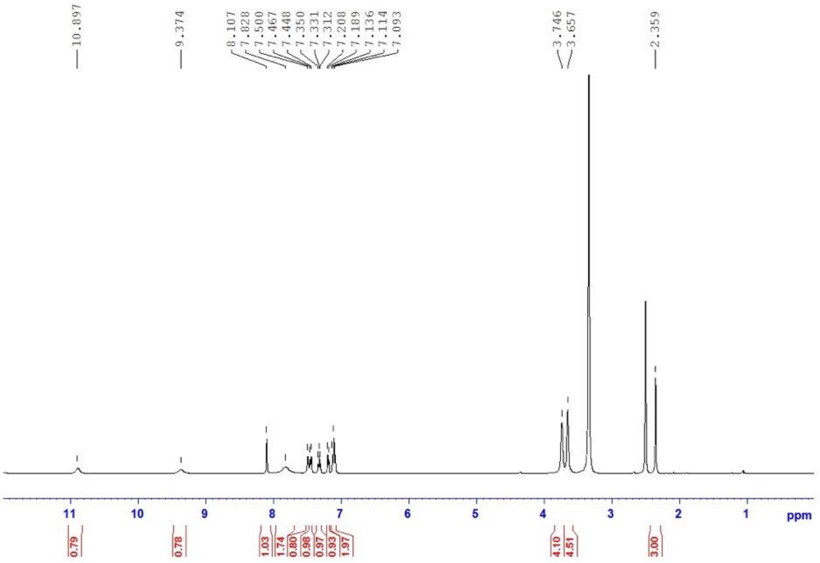

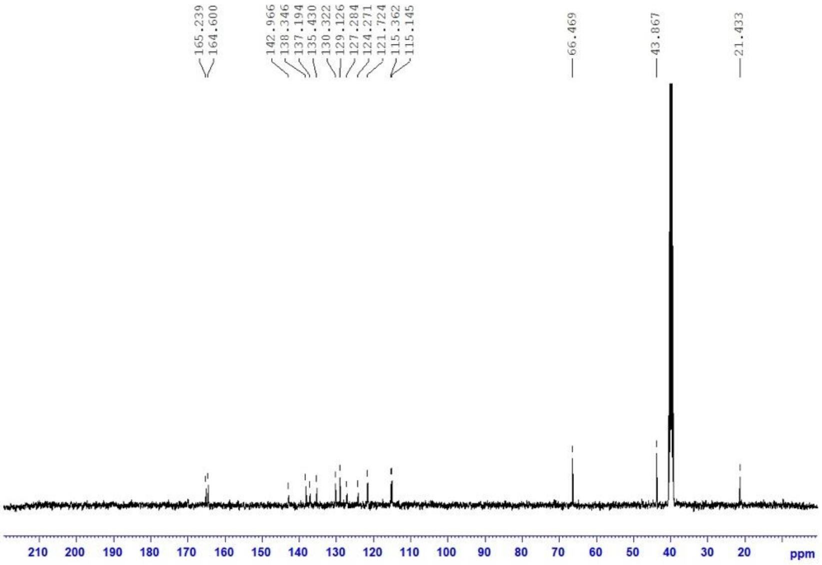

White powder (60 mg, 0.147 mmol, 46%); R_f = 0.31 (HEX/EtOAc, 3:1);

^{1}H NMR (400 MHz, DMSO-d_6) δ 10.89 (bs, 1H), 9.37 (bs, 1H), 8.11 (s, 1H), 7.81 (bs, 2H), 7.49 (t, *J*= 8Hz, 2H), 7.33 (t, *J*=7.6 Hz, 2H), 7.19 (d, *J*=7.6 Hz, 1H), 7.12 (t, *J*=8 Hz, 2H), 3.75 (bs, 4H), 3.66 (bs, 4H), 2.36 (s, 3H); ^{13}C NMR (100 MHz, DMSO-d_6): 165.2, 164.6, 142.9, 138.3, 137.2, 135.4, 130.3, 129.1, 127.3, 124.3, 121.8, 115.4, 115.2, 66.5, 43.9, 21.44 ppm. MS *m/z* (ESI$^+$) m/z: 408.2 [M+H] $^+$. MS *m/z* for $C_{21}H_{22}FN_7O$, (ESI$^+$) *m/z*: 430.1 [M+Na]$^+$, 837.3 [2M+Na]$^+$; (ESI$^-$) *m/z*: 406.2 [M-H]$^-$.

2. Antiviral assays—HIV-1

The antiviral activity of investigational compounds was evaluated by measuring the IC_{50} values against the HIV-1 wild-type reference strain NL4-3 in a TZM-bl cell line based phenotypic assay named BiCycle Assay.[2] The method includes a first round of infection in H9 cells, at 0.08 MOI in presence of serial dilutions of the compound in a 96-well plate. In each plate the reference compound, the mock control (uninfected cells) and the virus control was included. After 72 hours, 50 microliters of supernatants from each well were used to infect TZM-bl cells, which allow the quantitative analysis of HIV-1 infection by measuring the expression of the luciferase gene under the control of the HIV-1 LTR promoter. After 48 hours, dose-response curves were generated by measuring reporter gene expression in each well by using Bright-Glo Luciferase Assay (Promega) through the GloMax® Discover Multimode Microplate Reader (Promega). Relative luminescence units measured in each well were elaborated with the GraphPad PRISM software version 9 to calculate IC_{50} values.

Table S1. Antiviral activity and cytotoxicity of the synthesized compounds *in vitro* in a cell-based model.[a.]

Entry	Compound	IC_{50} ⚜M[b]	CC_{50} ⚜M[c]
1	**10 a**	NA	>100
2	**10b**	NA	>100
3	**10c**	NA	74
4	**13a**	NA	>100
5	**13b**	NA	>100
6	**14a**	NA	30
7	**14b**	NT	>100
8	**Raltegravir**	2.8 ± 1.7[d]	>100

[a]All experiments were conducted in H9 cells in duplicate in 3 independent experiments; [b]IC_{50} half-maximal compound concentration inhibiting the 50% of HIV-1 replication; [c]CC_{50}, half-maximal compound cytotoxic concentration, as determined by Cell-Titer Glo kit (Promega); [d]IC_{50} expressed in nM unit. NA = not active; NT = not tested, highly cytotoxic.

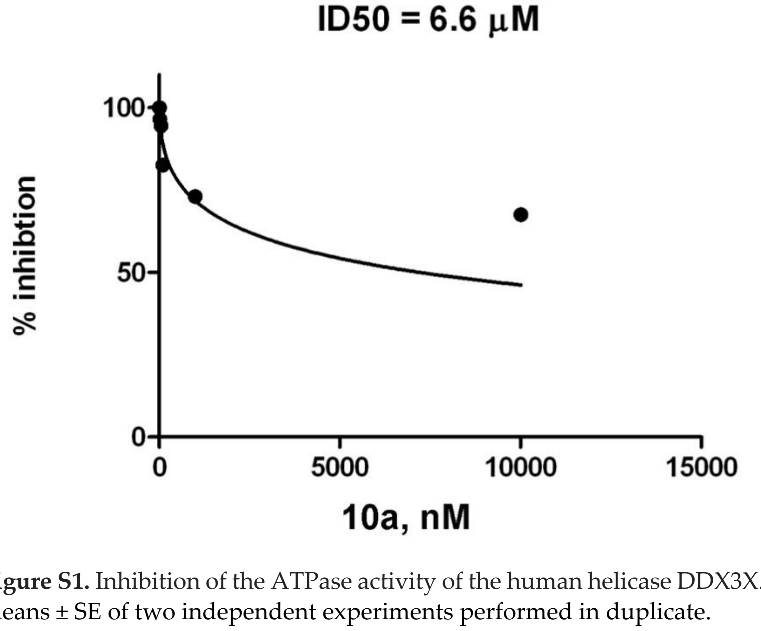

Figure S1. Inhibition of the ATPase activity of the human helicase DDX3X. Values indicate the means ± SE of two independent experiments performed in duplicate.
